# In Memoriam

**DOI:** 10.1093/ijnp/pyab019

**Published:** 2021-05-15

**Authors:** John R Mantsch, Gavin Bart, Ellen M Unterwald

**Affiliations:** 1 Florence Williams Professor and Chair, Department of Pharmacology & Toxicology, Medical College of Wisconsin; 2 Director, Professor of Medicine, Division of Addiction Medicine, Hennepin Healthcare, University of Minnesota Medical School; 3 Professor, Pharmacology, Director, Center for Substance Abuse Research, Lewis Katz School of Medicine, Temple University

**Figure:**
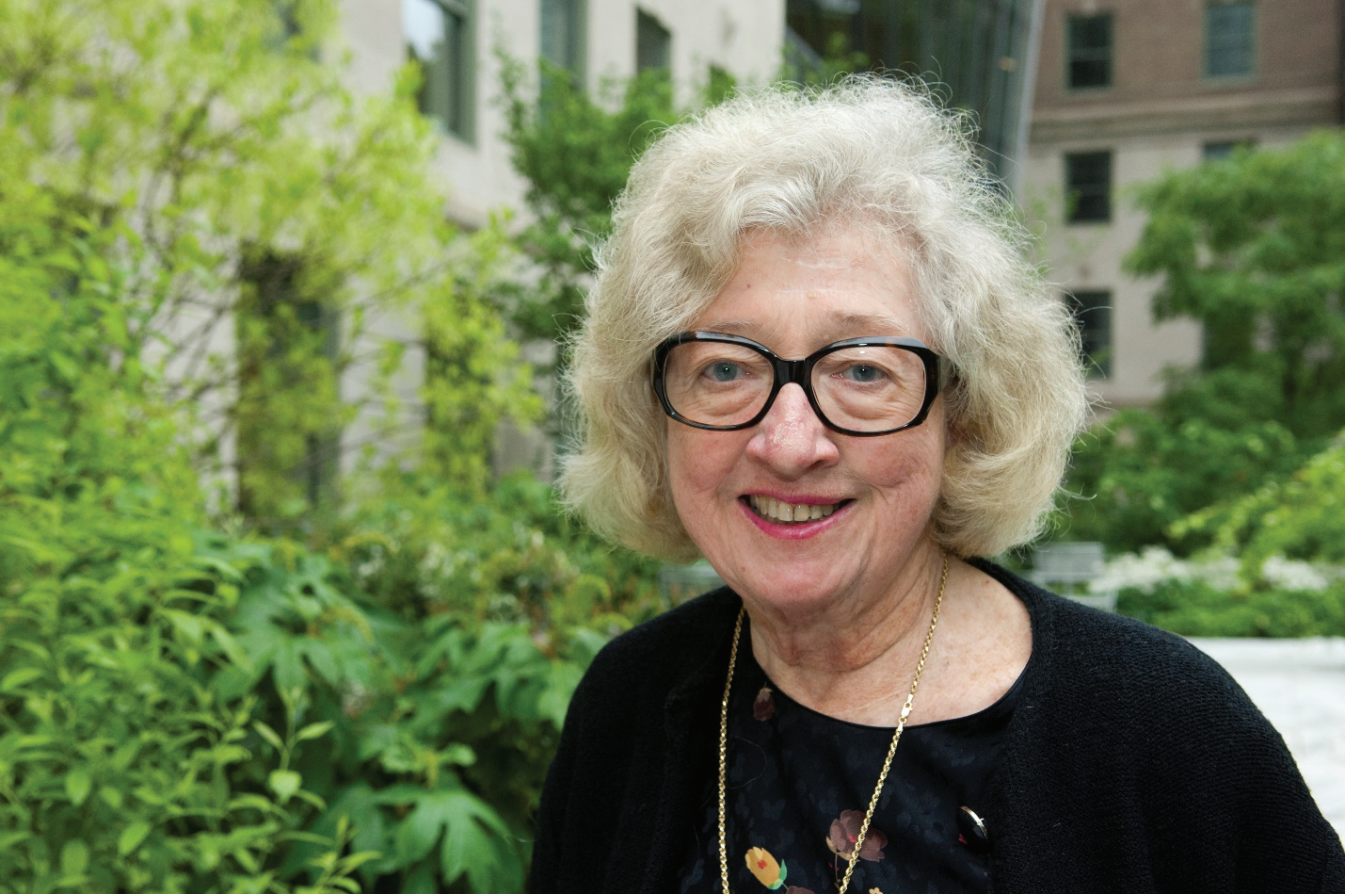
Dr. Mary Jeanne Kreek, M.D., Professor of Medicine. Photo credit: Zach Veilleux/The Rockefeller University

The fields of neuropsychopharmacology and medicine lost a pioneer and legend on March 27^th^, 2021 with the death of Mary Jeanne Kreek at the age of 84. Dr. Kreek, the Patrick E. and Beatrice M. Haggerty Professor and a senior attending physician at The Rockefeller University, was a world-renowned physician-scientist, a staunch advocate for those struggling with drug addiction, and a champion for women in science. She redefined translational research on addiction and was a driving force in guiding national drug policy and NIH research priorities. She worked tirelessly to destigmatize and change societal views on substance use disorders. Notably, Dr. Kreek established herself as a leader in science and medicine during a time when there were numerous obstacles for women. She shattered glass ceiling after glass ceiling, and empowered by her experience, Dr. Kreek stood as a role model and fierce supporter of the next generation of women scientists and physicians.

Dr. Kreek was born in Washington, DC in 1937. As a child she was stricken with paralytic polio, and as part of her recovery, she took up classical ballet and danced with a national ballet company for several years. Her experience with polio also reinforced her interest in science and medicine, for which she showed great aptitude at a young age. Indeed, when asked about her accomplishments, Dr. Kreek would often fondly cite her high school National Science Fair and Westinghouse Talent Search awards, even before mentioning her multitude of professional honors. Dr. Kreek was a very proud 1958 graduate of Wellesley College, where she studied chemistry and biology. She elected to attend medical school at the Columbia University College of Physicians and Surgeons and was one of only three women in her graduating class in 1962. As a student, Dr. Kreek’s research experience included work with the endocrinologist, Frederic Bartter, at the time the Chief of the National Institutes of Health Endocrine-Hypertension Branch.

In 1962, Dr. Kreek began a residency in internal medicine at the Cornell University New York Hospital Medical Center (now Weill Cornell Medicine) and, along with Dr. Marie Nyswander, she joined the research team of Dr. Vincent Dole at the Rockefeller Institute for Medical Research (now the Rockefeller University) in 1964. Together, the team established a program aimed at developing a medication to treat heroin addiction – at the time a growing urban crisis, especially in New York City. Work from the team was among the earliest to characterize heroin addiction as a metabolic disorder that includes alterations in brain function with behavioral consequences – a critical advance in understanding and humanizing addiction and a perspective that has been fundamentally transformative for guiding treatment. Much of the key observational work was conducted in active heroin users residing in the Rockefeller University Hospital. In 1966, the team published the first paper demonstrating that methadone can be used to treat heroin addiction in the Archives of Internal Medicine (now JAMA Internal Medicine) (Dole et al. 1966). Dr. Kreek and her colleagues found that that oral administration of the slow onset, long-acting synthetic opioid methadone, did not produce the intense euphoria that patients had experienced when they used heroin. More importantly, when patients were maintained on methadone, they did not experience the “high” associated with heroin use, nor did they experience the dysphoria associated with withdrawal. Primarily through the continued efforts of the team, by 1974 the FDA approved and established a framework for the use of methadone in the treatment of narcotic addiction. This work paved the way for the use of methadone and other related pharmacotherapies, such as buprenorphine, around the world and has saved and improved countless lives.

In 1975, when Professor Vincent Dole redirected his research efforts to the study of alcohol, Dr. Kreek was provided the opportunity to independently carry forward her work on opioid addiction, one of the first women to serve as a laboratory head at the Rockefeller University. That laboratory was eventually named the Laboratory of the Biology of Addictive Diseases. In 1983, with her promotion to Associate Professor, Dr. Kreek became only the fourth woman to receive tenure at Rockefeller. She earned the distinction of Full Professor in 1994 and received the Patrick E. and Beatrice M. Haggerty endowed professorship in 2004. As Dr. Kreek’s laboratory grew, so did the scope and breadth of her science. Recognizing that meaningful advances in treating drug addiction will require multidisciplinary collaborative research teams, state-of-the-art basic research platforms, and an effective framework for translation, she recruited scientists and clinician-researchers with backgrounds in psychiatry, clinical psychology, genetics, chemistry, molecular biology, behavioral neuroscience, and pharmacology (among other areas) and began translational research programs aimed at understanding how brain function is altered in substance use disorders, the genetic determinants of drug addiction, and how opioid effects on immune function could influence the risk for HIV and infections such as hepatitis B and C, and tuberculosis. By 1995, she was directing two NIH-funded research centers – one focused on neurobiology and a second focused on genetics. While many will understandably cite her work with methadone as her most significant contribution to science, Dr. Kreek’s scientific legacy extends well beyond this and includes tremendous advances in our understanding of gene variants that increase the risk for or protect against addiction as well as work exploring novel pharmacotherapies for alcohol and cocaine use disorders.

Dr. Kreek authored more than 500 research, review, and perspective papers. Her far-reaching research network extends across the globe. She received honorary degrees from Uppsala University, Tel Aviv University, and the University of Bologna and held appointments at Peking University and the Karolinska Institute. Dr. Kreek has directly mentored numerous scientists, clinicians, and policymakers and inspired many more through her leadership, which included prolific roles in many prominent scientific organizations and initiatives. In particular, she took immense pride in her involvement in the College (formerly Committee) on Problems of Drug Dependence (CPDD) and the International Narcotics Research Conference (INRC). She was a past president and executive board member in each organization and, in 1999, she received the Nathan B. Eddy Memorial Award for Lifetime Excellence in Drug Abuse Research from CPDD acknowledging her amazing contributions to addiction research. Many have described Dr. Kreek as a force of nature. It was only appropriate that the meeting at which she received this award in Acapulco Mexico was briefly disrupted by a magnitude 6.7 earthquake. The Eddy Award was one of many (too numerous to list them all) which included a Lifetime Achievement Award from the National Institute on Drug Abuse, the Betty Ford Award from The Association for Multidisciplinary Education and Research in Substance use and Addiction (AMERSA), the Marian W. Fischman Lectureship Award from the CPDD, the INRC Founders Lecture Award, alumni achievement awards from Wellesley and Columbia, and a Recognition Award for Research in Science of Addiction from the Executive Office of the President - Office of National Drug Control Policy.

Dr. Kreek was actively involved in research and continued to advocate for pharmacotherapy for opioid use disorder and destigmatizing drug addiction until her death. In a recent interview, she expressed frustration that opioid overdose rates have been increasing precipitously in the U.S. while access to treatment remains limited and efforts to educate clinicians and policymakers continue to be met with resistance. At the same time Dr. Kreek also relayed satisfaction that her work advanced the perspective that opioid addiction is a brain disease and that her efforts related to methadone, buprenorphine, and other pharmacotherapies have provided hope and increased the quality of life for so many. She also expressed excitement for the younger generation of scientists and physicians who will continue to carry forward her legacy and in particular, the many women scientists whom she has empowered and inspired. As many will attest, despite her stature and busy schedule, Dr. Kreek was always eager to take the time to give advice and encouragement to young scientists. Recently, Dr. Kreek was asked to write about her experience as a woman in science. She agreed to do so, but only with the condition that three of her trainees could share their own experiences as women in science well (Kreek et al. 2021). Thus, as she has always done, Dr. Kreek used her hard-earned influence to provide a voice for those who needed to be heard.

Dr. Kreek will be deeply missed by her colleagues, her many collaborators, and her trainees. For all that she has accomplished, Dr. Kreek’s family was always her greatest source of pride. Her husband of 48 years, Dr. Robert Schaefer Sr., who was a gastroenterologist, associate professor at Weill Cornell Medicine, and occasional scientific collaborator died in 2018. She leaves behind her daughter Dr. Esperance Schaefer and son Robert Schaefer, Jr., along with their spouses and four grandchildren.

Dr. Kreek’s enthusiasm, energy, passion for science, and dedication to improving the lives of those struggling from substance use disorders will be greatly missed. As a scientist, mentor, advocate, and policy influencer, she refused to be ignored. As the current opioid crisis worsens, Dr. Kreek’s death comes at a time when her leadership, voice and guidance are needed more than ever. It is our hope that those whom she has inspired with her words and actions are prepared to carry out her legacy and to advocate for compassionate policies, access to treatment, and humanization of those suffering from the disease of addiction.
